# Mesenchymal stem/stromal cells-derived extracellular vesicles as a potentially more beneficial therapeutic strategy than MSC-based treatment in a mild metabolic osteoarthritis model

**DOI:** 10.1186/s13287-023-03368-7

**Published:** 2023-05-24

**Authors:** Kelly Warmink, Jaqueline L. Rios, Suzy Varderidou-Minasian, Marta Torres-Torrillas, Devin R. van Valkengoed, Sabine Versteeg, Niels Eijkelkamp, Harrie Weinans, Nicoline M. Korthagen, Magdalena J. Lorenowicz

**Affiliations:** 1 Regenerative Medicine Center, Uppsalalaan 8, 3584 CT Utrecht, The Netherlands; 2 Department of Orthopedics, University Medical Center Utrecht, PO Box 85500, 3508 GA Utrecht, The Netherlands; 3 Center for Molecular Medicine, University Medical Center Utrecht, Universiteitsweg 100, 3584 CG Utrecht, The Netherlands; 4 Bioregenerative Medicine and Applied Surgery Research Group, Department of Animal Medicine and Surgery, CEU Cardenal Herrera University, CEU Universities, Valencia, Spain; 5 García Cugat Foundation CEU-UCH Chair of Medicine and Regenerative Surgery, CEU Cardenal Herrera University, CEU Universities, Valencia, Spain; 6 Center for Translational Immunology, University Medical Center Utrecht, Utrecht University, PO Box 85090, 3508 AB Utrecht, The Netherlands; 7 Department of Biomechanical Engineering, TU Delft, Mekelweg 2, 2628 CD Delft, The Netherlands; 8 Biomedical Primate Research Centre, Lange Kleiweg 161, 2288 GJ Rijswijk, The Netherlands

**Keywords:** Mesenchymal stem/stromal cells, Extracellular vesicles, Cartilage regeneration, Inflammation, Osteoarthritis, Rat high fat diet groove model

## Abstract

**Background:**

Mesenchymal stromal/stem cells (MSCs) and MSC-derived extracellular vesicles (MSC-EVs) hold promise as a disease modifying treatment in osteoarthritis (OA). Obesity, and its associated inflammation, contribute to OA development and metabolic OA represents a specific and significant group of the OA patient population. Given their immunomodulatory properties, MSC and MSC-EVs are especially interesting for this group of patients as a therapeutic option. Here, we were the first to compare the therapeutic efficacy of MSCs and MSC-EVs in a mild OA model taking these metabolic aspects into consideration.

**Methods:**

Male Wistar-Han rats (Crl:WI(Han) (n = 36) were fed a high fat diet for 24 weeks, with unilateral induction of OA by groove surgery after 12 weeks. Eight days after surgery rats were randomized in three treatment groups receiving MSCs, MSC-EVs or vehicle injection. Pain-associated behavior, joint degeneration, and local and systemic inflammation were measured.

**Results:**

We demonstrated that despite not having a significant therapeutic effect, MSC-EV treatment results in lower cartilage degeneration, less pain behaviour, osteophytosis and joint inflammation, than MSC treatment. Suggesting that MSC-EVs could be a more promising therapeutic strategy than MSCs in this mild metabolic OA model.

**Conclusion:**

In summary, we find that MSC treatment has negative effects on the joint in metabolic mild OA. This is an essential finding for the significant group of patients with metabolic OA phenotype, and might help to understand why clinical translation of MSC treatment shows varying therapeutic efficacy thus far. Our results also suggest that MSC-EV-based treatment might be a promising option for these patients, however MSC-EV therapeutic efficacy will need improvement.

**Supplementary Information:**

The online version contains supplementary material available at 10.1186/s13287-023-03368-7.

## Introduction

Osteoarthritis (OA) is the most common form of arthritis and affects 16% of the worldwide population [1]. The disease is marked by a gradual degeneration of the joint accompanied by low-grade inflammation, which result in chronic pain and immobility [2]. Currently, there is no effective disease-modifying cure for OA [3].

Recently, mesenchymal stromal/stem cells (MSCs) have emerged as a new promising disease modifying therapy for OA. Due to their immunomodulatory and regenerative capacities MSCs have potential to restore the imbalance between anabolic and catabolic processes in an OA joint [4–6]. Intra-articular injections with human MSCs show little adverse events in clinical trials.

Although promising as treatment option, the fate of MSCs in vivo and the molecular mechanism underlying their beneficial effects remain unclear. Increasing evidence suggests that the therapeutic efficacy of MSCs depends on paracrine signaling [7, 8], and more recently their therapeutic potential has been attributed to the secretion of extracellular vesicles (EVs) [9–11]. EVs are membrane structures, which exert many of their functions as an intercellular shuttle, carrying cargo such as protein and RNA to be transferred from one cell to another [12].

MSCs and MSC-EVs have very similar immunomodulatory capacities [13]. Both convert pro-inflammatory M1-like macrophages into an anti-inflammatory M2-like phenotype [14]. Recently, we have shown that MSC-EVs inhibit tumor necrosis factor (TNF) stimulated inflammatory responses in chondrocytes from OA patients and promote cartilage regeneration in vitro [15]. Importantly, MSC-EVs have regenerative properties in vivo. They promote cutaneous wound healing [9], diminish myocardial ischemia/reperfusion injury, and stimulate regeneration in osteoarthritis models [16–20]. Thus, MSC-EVs have a great potential to serve as an off-the-shelf, cell free therapy for OA.

Obesity is one of the biggest risk factors for OA, and the metabolic OA phenotype represents a specific part of the patient population [21, 22]. The high fat diet groove rat model mimics metabolic OA, by inducing a low-grade systemic inflammation and slowly developing, mild or early stage joint degeneration [23–25]. The involvement of both inflammation and degeneration makes it a unique OA model and ideal to study regenerative and anti-inflammatory capacities of MSCs and MSC-EVs in vivo. Therefore, purpose of this study was to compare therapeutic efficacy of MSCs and MSC-EVs in this model, representing an early stage of metabolic OA. There are few studies that compare MSC and MSC-derived EVs in the same model, and there are no studies, as of yet, that compare both treatments in a mild OA model with disturbed metabolic context, even though this represents a large subset of the human OA patient population [26].

## Methods

### Animals and OA model

36 healthy male, 12 week old Wistar-Han rats [Crl:WI(Han) Charles-River, Sulzfeld, Germany] were fed ad libitum high fat diet (E15742-34; 60 kcal% fat, 20 kcal% carbohydrates and 20 kcal% protein, Sniff Bio-Services, Soest NL) for 24 weeks to induce metabolic dysregulation. In week 12, all rats received unilateral groove surgery in random order, where local cartilage damage was induced in the right knee joint by making longitudinal grooves on the femoral condyles and trochlea to induce OA, as described previously [23]. The contralateral knee joint was not surgically damaged as internal control. Analgesia (0.01 mg/kg buprenorphine) was injected subcutaneously before, and 6–8 h after surgery. General anaesthesia (isoflurane) was used during the surgical procedure and animals were allowed to move freely directly after surgery. Eight days after surgery, animals were randomized by weight into three groups (n = 12 per group), using digitally generated random numbers, receiving 25 μL intra-articular injection in the grooved joint with either phosphate-buffered saline (PBS, vehicle group), 2 × 10^6^ MSCs in PBS (MSCs group) or EVs (7.77 × 10^7^ particles) derived from 2 × 10^6^ MSCs in PBS (MSC-EVs group). The extracellular vesicles group received five of these doses with 5-day intervals. In week 24 of the experiment, animals were euthanized by aorta puncture under general anaesthesia (isoflurane), and tissues were harvested for further evaluation. Rats were housed per two in open polycarbonate cages (Type IV) under a 12:12 light–dark cycle, provided with nesting material, nest box and solid wood block. Group allocation was blinded and animal order was randomized during the experiment, all measurements and data analysis. No animals were excluded during the experiment. A priori exclusion criteria were; bacterial infection or signs of severe discomfort (joint unloading and/or > 15% weight loss). Animals were weekly monitored and weighed. Experiment study protocol was approved by the Utrecht University Medical Ethical Committee for animal studies (licence AVD115002016688) and was in compliance with European Community specifications regarding the use of laboratory animals. Study protocol was based on previous research [23–25], and was not (pre)registered.

### Human bone marrow derived mesenchymal stem cell isolation and culture

The MSCs used in this study are classified as Advanced therapy medicinal product (ATMP) and manufactured in the GMP-licensed Cell Therapy Facility, Department of Clinical Pharmacy of the University Medical Center Utrecht. Briefly, bone marrow aspirates were obtained from third-party healthy donors as approved by the Dutch Central Committee on Research Involving Human Subjects (CCMO, Biobanking bone marrow for MSC expansion, NL41015.041.12). The bone marrow donor or the parent or legal guardian of the donor signed the informed consent as required by the CCMO. Bone marrow was separated using a density gradient centrifugation (Lymphoprep, Axis Shield, Dundee, United Kingdom). MSCs were isolated by plastic adherence and expanded using the MC3 systems and α-minimal essential medium (α-MEM) with l-glutamine (Macopharma, Tourcoing, France) supplemented with 5% human platelet lysate (PL) and 3.3 IU/mL heparin (Leo Pharma, Ballerup, Denmark) up to passage 3 [27]. Characterization of MSCs fitted the internationally defined minimal criteria for these cells [27, 28]. The ATMP MSCs used in this study were cultured for additional passages (passage 4–7) in α-MEM (Gibco Invitrogen, Carlsbad, CA, USA) supplemented with 5% human platelet lysate (PL), 100 U/mL penicillin and 100 μg/mL streptomycin (Gibco Invitrogen) and 10 U/mL heparin and maintained at 37 °C and 5% CO_2_. The PL was depleted from PL-derived EVs by overnight centrifugation at 100,000×*g*.

### Extracellular vesicle isolation

EVs were isolated from the MSC culture medium as described previously [29]. In brief, MSCs were cultured for 48 h in medium depleted from EVs by overnight centrifugation at 100,000×*g* in SW32Ti rotor (Beckman Coulter, Brea, USA). To isolate the MSC-EVs, the conditioned medium was subjected to differential centrifugation. First, cells were removed by two centrifugations at 200×*g* for 10 min. Collected supernatant was subsequently centrifuged two times at 500×*g* for 10 min, followed by 10,000×*g* for 45 min. Vesicles were finally pelleted by ultracentrifugation at 100,000×*g* for 16 h in SW32Ti rotor (Beckman Coulter) followed by washing in PBS and pelleting in SW60 rotor (Beckman Coulter). MSC-EVs were stored in PBS at − 80 °C until use.

### Sucrose density gradient and western blotting

Extracellular vesicles isolated by differential centrifugation were suspended in 250 μL PBS-2.5 M sucrose, loaded in a SW60 tube and overlaid with 15 successive 250 mL layers of 20 mM Tris pH 7.4 containing decreasing concentrations of sucrose (from 2 to 0.4 M). Tubes were centrifuged for 16 h at 200,000×*g* at 4 °C. Fractions of 250 μL were collected and sucrose density was measured using a refractometer. Fractions were mixed 1:1 with Laemmli sample buffer and incubated for 5 min at 95 °C, followed by SDS-page and Western blotting analysis using standard procedures. In brief, proteins were transferred to polyvinylidene difluoride (PVDF) membrane (Millipore) and incubated with the following antibodies: mouse anti-CD9 (1:1000; Biolegend), mouse anti-CD63 (1:1000; Abcam). Membranes were washed, incubated with appropriate peroxidase-conjugated secondary antibodies and developed by SuperSignal West Dura or Femto Extended Duration Substrate (ThermoFisher).

### Nanoparticle tracking analysis

Size distribution and quantification of isolated MSC-EVs was determined by nanoparticle tracking analysis using NanoSight NS500 instrument (Malvern Instruments Ltd, Malvern, UK), equipped with sCMOS camera. Data was analyzed with the nanoparticle tracking analysis software version 3.1. (build 3.1.54), with detection threshold set to 5, and blur and Max Jump Distance set to auto. Samples were diluted 100-, 300-, 500-, 700- and 1000-fold with PBS to reach optimal concentration for instrument linearity. Readings were taken at a camera level set to 13 and with manual monitoring of temperature.

### Blood measurements

In week 0 and 24, blood was obtained from the tail vein of 16-h fasted rats and collected in a lithium heparin plasma tube (BD Microtainer, BD, USA), and in a clot activator serum tube (BD Microtainer, BD, USA). Tubes were put on ice and centrifuged (15 min, 2000×*g*) within 30 min after collection. Plasma and serum aliquots were stored at − 80 °C until analysis. Plasma glucose and triglyceride levels (University Veterinary Diagnostic Laboratory of the Utrecht University) and serum insulin levels were determined (EZRMI-13 K, Millipore, Amsterdam, The Netherlands). Insulin resistance index HOMA-IR (Homeostatic Model Assessment for Insulin Resistance) was calculated according to Matthews et al. [30]. Serum cytokines and chemokines were measured by rat 27 multiplex assay (Millipore, Darmstadt, Germany) performed at the MultiPlex Core Facility of the University Medical Center Utrecht, The Netherlands. Out of range values were excluded from analysis.

### Pain-associated behaviour

Dynamic weight bearing (DWB) and Von Frey methods were used to monitor pain-associated behaviours [31]. All behavioural measurements and data analysis were done blinded and in random order. Two baseline measurements were obtained 1 week before surgery. Rats were measured 5 days after surgery and 1 day before and after each injection, and then every other week until endpoint. Von Frey method was used to assess mechanical hypersensitivity by applying von Frey hairs to the plantar surface of the hind paws. Rats were placed in a cage with a wire mesh floor and allowed to acclimate for 15 min. The hair force was increased or decreased according to the response and the 50% paw withdrawal threshold was calculated using the up-and-down method [31]. DWB was performed in a 22 × 22 cm plexiglass chamber (30 cm high) with a pressure sensitive sensor floor (setup from Bioseb, module version 1.4.2.98; Boulogne, France), where rats could move freely for 5 min. A blinded observer compared animal placement on video with sensor activation and determined the amount of weight placed on each separate paw. A pressure zone was considered valid when the following parameters were detected [32]: ≥ 2 g on one captor with a minimum of 3 adjacent captors recording ≥ 1 g for more than 0.5 s, and time spend rearing was excluded.

### Micro computed tomography scans

Micro computed tomography (µCT) scans were made at week 0, 12 and 24 under anesthesia (2% isoflurane) using a Quantum FX CT scanner (PerkinElmer, Waltham, MA). Both hind limbs were positioned in extension scanned for 3 min at an isotropic voxel size of 42 μm, voltage of 90 kV, current of 180 μA and a field of view of 21 mm. ImageJ software (ImageJ, 1.47v, NIH, Bethesda, USA) was used for analyses. In serial 2D scans the number of osteophytes, bone cysts and other abnormalities were counted.

In addition, the bone was segmented using a local threshold algorithm (ImageJ, Bernsen algorithm, using radius 5) to evaluate tibial subchondral plate thickness (µm), trabecular bone thickness (µm) and volume fraction [33]. The trabecular bone volume fraction (BV/TV) was calculated by the ratio of trabecular bone volume (BV, in mm^3^) and endocortical tissue volume (TV, in mm^3^). Regions of interest were manually drawn in coronal orientation in 90 slides, starting at the back of the knee from the point where the medial and lateral compartments of the tibial epiphysis unite and moving to the front of the joint.

### Histology

Harvested knee joints were fixed in neutral buffered formaldehyde 4% for 48 h and then decalcified in 0.5 M EDTA set to pH 7.0 with NaOH for 6 weeks. Every week the samples were re-fixed in formaldehyde 4% for 24 h. The decalcified tissue was dehydrated for 50 h in a fully enclosed tissue processor (Leica biosystems, Amsterdam, Netherlands) using a series of 70–100% ethanol, cleared in xylene and finally infiltrated and embedded in paraffin. Coronal 5 µm sections were made at 200 μm intervals over a total of 1200 μm and stained with Weigert’s Hematoxylin, Fast Green and Safranin-O. The joint degeneration was evaluated using the OARSI histopathology score for rats [34], where the 3 worst sections from each joint were scored in a blinded and randomized order. The total OARSI score is the sum of the following subscores: cartilage degeneration (0–60), calcified cartilage and subchondral bone damage (0–5), osteophyte size (0–4) and synovial membrane inflammation (0–4). In the cartilage degeneration, all four cartilage compartments (tibia and femur, medial and lateral) are included, each having a score of 0–15. On the femur, damage observed due to surgical applied grooves was not included when scoring the cartilage damage, but the direct adjacent cartilage was included.

Immunohistochemistry staining was performed on knee joint sections for Inducible nitric oxide synthase (iNOS) and Matrix metalloproteinase 13 (MMP-13). For both types of staining, slides were blocked for endogenous peroxidase using 0.3% H_2_O_2_ for 10 min at room temperature. Antigen retrieval was performed for iNOS using 0.1% pepsin (Sigma, Saint Louis, USA) in 0.02 M HCl solution for 30 min at 37 °C. Antigen retrieval for MMP-13 was done using 0.01 M citrate buffer: Tri-sodium Citrate dihydrate (Merck, Darmstadt, Germany) in demi water, adjusted to pH 6.0 with 1 M Citric Acid (Sigma-Aldrich, Saint Louis, USA) for 30 min at 70 °C. For both types of staining, all slides were blocked in 5% PBS-BSA for 30 min at room temperature. Primary iNOS antibody incubation was done overnight at 4 °C with 0.4 µg/mL iNOS mouse monoclonal antibody (SC-7271, Santa Cruz Biotechnology, Santa Cruz, Dallas, TX, USA) or with 0.4 µg/mL normal mouse IgG1 as isotype control (sc3877, Santa Cruz, Dallas, TX, USA). Primary MMP-13 antibody incubation was done overnight at 4 °C with 2 µg/mL MMP-13 mouse monoclonal antibody (SC-515284, Santa Cruz Biotechnology, Santa Cruz, Dallas, TX, USA) or with 2 µg/mL normal mouse IgG1 as isotype control (sc3877, Santa Cruz, Dallas, TX, USA). All antibodies were diluted in 5% PBS-BSA. The next day, slides were incubated with anti-mouse HRP (Envision, Dako, CA, USA) for 30 min at room temperature and subsequently incubated with liquid DAB + 2-component system (Agilent, Santa Clara, CA, USA) for 5 min. Sections were counterstained with hematoxylin, dehydrated, cleared in xylene and cover slipped with Eukitt mounting medium (Sigma-Aldrich, Saint Louis, USA). For each structure of interest (cartilage in case of MMP-13 and synovium in case of iNOS) three images per joint were captured at a 10× magnification. iNOS and MMP-13 staining was quantified in imageJ (1.47v, Bethesda, MD, USA), the region of interest (ROI) was manually drawn in each image. Images were converted to 8-bit and colour deconvolution with vector set to “H DAB” was run [35]. The brown colour-separated image was used to determine the percentage area of positive staining using the same threshold setting for every image of the respective tissue.

### Statistics

Sample size was determined by power analysis, using β = 0.2, α = 0.05, σ = 2.1 (based on [25]) and a difference of 3 points in the total OARSI score (primary outcome measure) was considered a clinically relevant difference (effect size = 1.43). One extra animal per group was included to compensate for potential loss of animals, resulting in 12 animals per group. Statistical analysis was performed using Prism (v7.04, GraphPad Software, La Jolla, CA, USA) and IBM SPSS software (v25.0, IBM SPSS Inc., Chicago, IL, USA). Normality was tested with the Shapiro–Wilk’s W test and checked graphically. For normally distributed outcomes one-way ANOVA with Tukey’s multiple comparisons test was used. For non-normally distributed outcomes Kruskal–Wallis with Dunn’s multiple comparisons test was used. Longitudinal data was tested using mixed models. Correlations between outcomes were tested using simple linear regression. To check if variances were equal for the body mass at week 11 and week 24 Barlett’s test was used. *p* values ≤ 0.05 were considered statistically significant. Graphs represent mean ± 95% confidence interval, *p* values in graphs are reported with asterisks where *p* ≤ 0.05 is *, *p* ≤ 0.01 is **, *p* ≤ 0.001 is *** and *p* > 0.05 is not significant (NS). Observers were always blinded with respect to group divisions while performing the measurements in a randomized order.

## Results

### MSC but not MSC-EV treatment promotes cartilage destruction and synovial inflammation in a rat high fat diet mild OA model

**Fig. 1:**
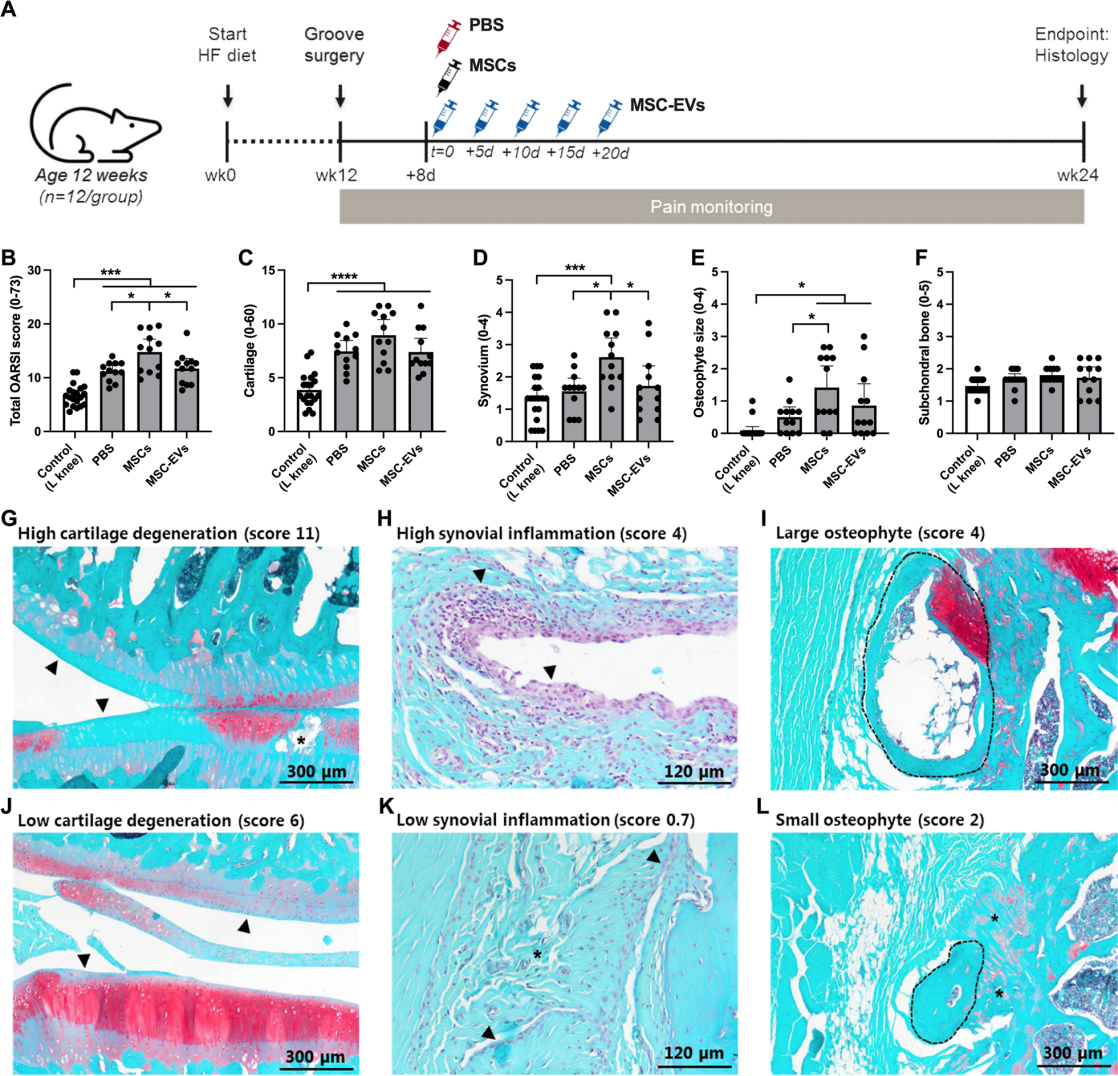
MSCs but not MSC-EV treatment promotes cartilage destruction and synovial inflammation. **A** Schematic overview of this animal study **B** Total OARSI score as sum of OARSI subscores: **C** cartilage degeneration, **D** synovial inflammation, **E** osteophyte size and **F** calcified cartilage and subchondral bone. **G**–**L** Representative safranin-O histology images of the range of joint degeneration in this OA model. **G** High level and **J** low level cartilage degeneration, arrows indicate loss of proteoglycans, asterisks indicate cartilage damage. **H** High level and **K** low level of synovial inflammation, arrows indicate synovial lining, asterisks indicate slight proliferation of the sub lining tissue. **I** High and **L** low osteophyte size, dotted lines encircle bony spurs, asterisks indicate fibrocartilage formation. No animals or data points were excluded from these analyses, n = 12 per group. Graphs represent mean ± 95% confidence interval, *p* values in graphs are reported with asterisks where *p* ≤ 0.05 is *, *p* ≤ 0.01 is **, *p* ≤ 0.001 is *** and *p* > 0.05 is not significant

To evaluate the therapeutic efficacy of bone marrow derived MSCs and bone marrow derived MSC-EVs in a mild OA model with disturbed metabolic context, MSCs and MSC-EVs were injected intra-articular, 8 days after surgery (Fig. 1A). The MSC-EVs used in this study were isolated by a well-established differential centrifugation method with the final MSC-EVs preparation pelleted by ultracentrifugation at 100,000×*g*. Isolated MSC-EVs were positive for exosomal markers such as CD9 and CD63 and were on average 125 nm in size (Additional file 1: Fig. S1 and [15]).

Cartilage destruction and synovial inflammation are the main histological features of OA, therefore, the effect of both treatments on joint degradation was evaluated using Fast green/Safranin-O staining. Joint degradation was scored using the OARSI histopathology score for rats [34]. The total OARSI score was higher in the right grooved knees in all three treatment groups, compared to the ungrooved control left knees (Fig. 1B) confirming the clear (but mild) development of OA in this model. Rats treated with MSC-EVs had statistically indistinguishable overall joint degradation compared to vehicle (PBS) treated rats, both having an average total OARSI score of ~ 12. In contrast, the MSCs treated group had a significantly higher total OARSI score (~ 15), indicating that MSCs unexpectedly aggravated joint degradation. The cartilage degeneration subscore for rats treated with MSCs demonstrated a trend of increased cartilage degradation, however this was not statistically significant (Fig. 1C, G, J).

In OA pathology, synovial inflammation is a major determinant of local immune activation, and it is the primary target of most immunomodulatory treatments. Importantly, rats treated with MSCs had elevated synovial inflammation, which was not the case for MSC-EV and vehicle treated animals, where the level of inflammation was moderate and resulted from HF diet (Fig. 1D, H, K). Also, the osteophyte size subscore was significantly increased in rats treated with MSCs and similar between vehicle and MSC-EV treated rats, corroborating the negative effect of MSCs on joint homeostasis (Fig. 1E, I, L). The subchondral bone scored low in all groups and showed no differences between groups (Fig. 1F). Altogether, these data indicate that MSC treatment had a clear negative influence on joint health, most strongly affecting synovial inflammation and osteophyte formation, while MSC-EVs had no significant therapeutic effect on OA in this model.

### MSC treatment but not MSC-EV treatment increases osteophyte volume

**Fig. 2:**
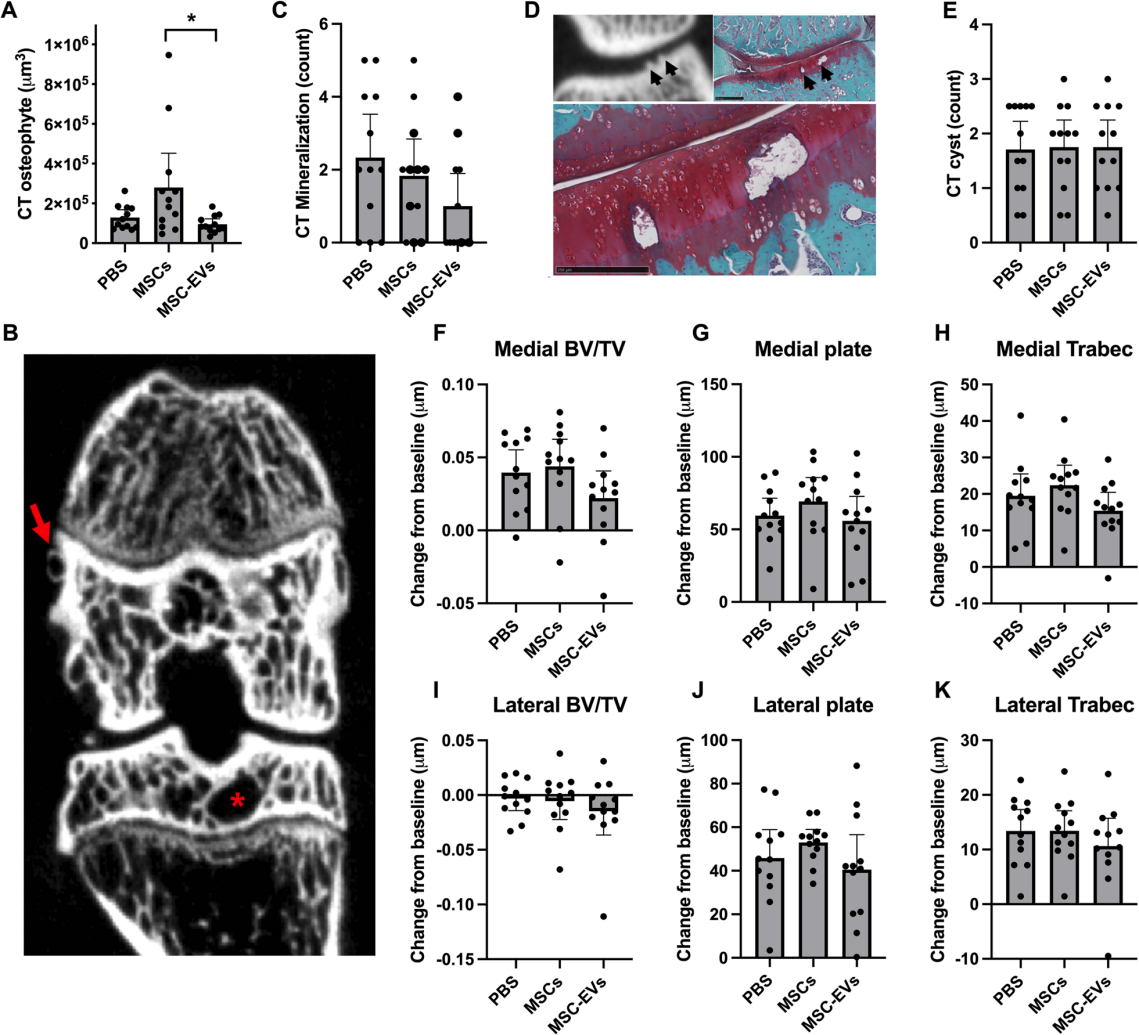
MSC treatment but not MSC-EV treatment increases osteophyte volume. **A** Total osteophyte volume measured on µCT. **B** Example of a µCT scan, where the red arrow indicates an osteophyte and the red asterisk indicates a cyst. **C** Number of cartilage mineralizations counted on the tibia plateau and **D** example of a cartilage mineralization as observed on µCT and safranin-O histology staining, arrows indicate mineralizations. **E** Number of cysts counted in the total joint. **F** Medial and **I** lateral tibial trabecular bone volume fraction, expressed as µm change from baseline. **G** Medial and **J** lateral tibial subchondral plate thickness, expressed as µm change from baseline. **H** Medial and **K** lateral tibial trabecular bone thickness, expressed as µm change from baseline. No animals or data points were excluded from these analyses, n = 12 per group. Graphs represent mean ± 95% confidence interval, *p* values in graphs are reported with asterisks where *p* ≤ 0.05 is *, *p* ≤ 0.01 is **, *p* ≤ 0.001 is *** and *p* > 0.05 is not significant

The formation of bony spurs, or osteophytes is a key clinical feature for the diagnosis of OA. In this study, we used µCT to evaluate the effect of MSCs and MSC-EVs treatment on osteophyte volume and other abnormalities such as bone cysts or cartilage mineralization. We observed a higher total osteophyte volume in the MSCs treated group (Fig. 2A, B) compared to MSC-EVs and vehicle treated rats, which corresponds to the negative effects of MSCs treatment detected by the histological analysis described above. Cartilage mineralization was present in 9 out of 12 animals in the vehicle and MSC groups and in 5 out of 12 animals in the MSC-EVs treated group (Fig. 2C, D; *p* = 0.15). Other type of abnormalities that may co-occur with OA, such as number of cysts (Fig. 2B, E), tibial epiphyseal bone volume fraction (Fig. 2F, I), tibial subchondral plate thickness (Fig. 2G, J), or tibial epiphyseal trabecular thickness (Fig. 2H, K) had changed from baseline, but were not affected by treatment.

### Increase in pain-associated behaviour in rats with mild OA is induced by MSCs but not MSC-EVs

**Fig. 3:**
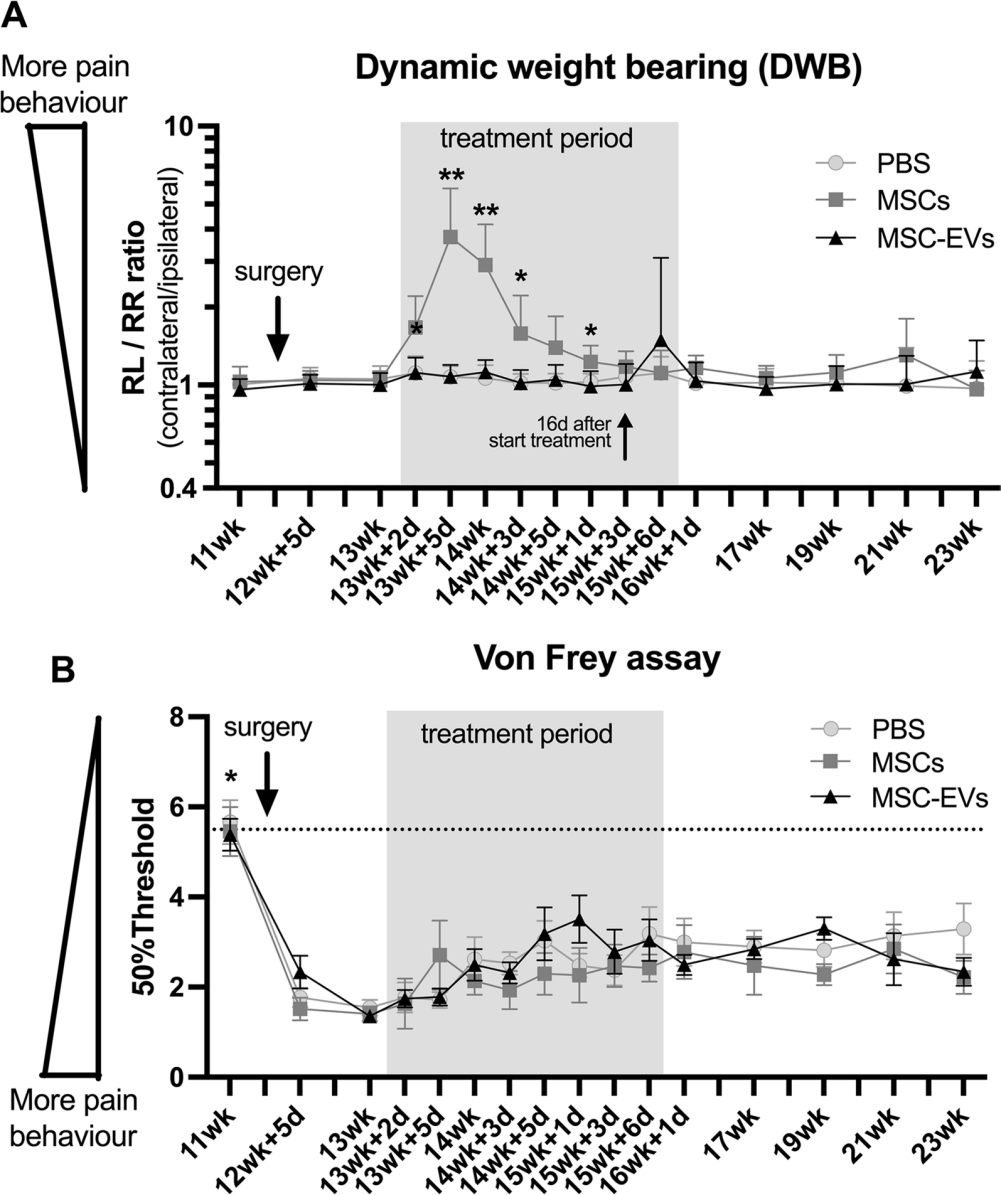
Increase in pain in rats with mild OA is induced by MSCs but not MSC-EVs. **A** Rear left/rear right (RL/RR) ratio measured on dynamic weight bearing (DWB) and **B** 50% paw withdrawal threshold measured on von Frey. No animals or data points were excluded from these analyses, n = 12 per group. Graphs represent mean ± 95% confidence interval, *p* values in graphs are reported with asterisks where *p* ≤ 0.05 is *, *p* ≤ 0.01 is **, *p* ≤ 0.001 is *** and *p* > 0.05 is not significant

In human OA patients, pain is one of the most prominent clinical symptoms. Here, we measured the effect of MSC and MSC-EV treatment on pain-associated behaviour in our mild OA model, using dynamic weight bearing (DWB) and Von Frey methods. Groove surgery did not affect the weight distribution between rear left (RL) and rear right (RR) limb (Fig. 3A). However, the RL/RR ratio increased 1 day after intra-articular injection with MSCs, indicating a shift in weight bearing away from the MSCs treated (right) limb to the untreated (left) limb. The RL/RR ratio remained higher compared to vehicle and MSC-EVs groups until 16 days after the MSCs injection. Groove surgery induced mechanical hypersensitivity as measured by Von Frey that persisted during the complete time course of experiment (Fig. 3B, *p* < 0.047 at all time points). However, no differences were observed between the three treatment groups (fixed treatment effect, *p* = 0.65), indicating the pain-associated behaviour induced by MSC treatment was stimulus-independent, spontaneous pain-associated behaviour.

### Systemic inflammation markers and joint MMP-13 levels are lowest in MSC-EV treated animals

**Fig. 4:**
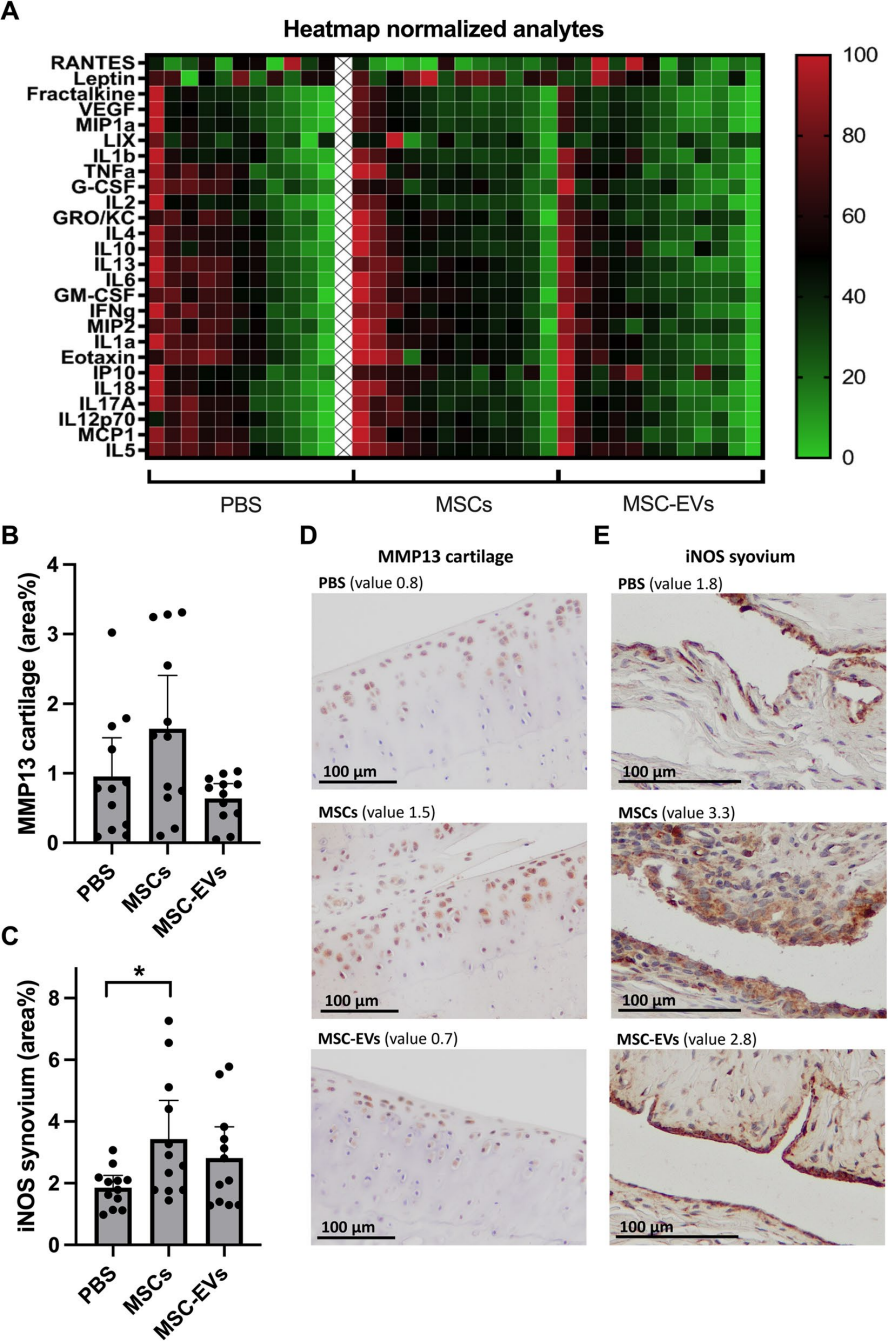
Systemic inflammation markers and joint MMP-13 are lowest in MSC-EV treated animals. **A** Heatmap showing 26 analytes normalized on a 0–100 scale, animals/columns are grouped by PBS, MSC or MSC-EV treatment. **B** percentage positive MMP-13 staining in the cartilage and **C** percentage positive iNOS staining in the synovium. Representative images showing staining intensity per group for **D** MMP-13 and **E** iNOS. The chosen images depict values close to the group mean. One animal was excluded from multiplex analysis due to out of range values (PBS n = 11; MSC n = 12; EV n = 12). EGF is not shown due to many out of range values (see full data set at Additional file 1: Fig. S2). No animals or data points were excluded from iNOS and MMP-13 analyses, n = 12 per group. Graphs represent mean ± 95% confidence interval, *p* values in graphs are reported with asterisks where *p* ≤ 0.05 is *, *p* ≤ 0.01 is **, *p* ≤ 0.001 is *** and *p* > 0.05 is not significant

Our OA model has a systemic metabolic and inflammatory component introduced by the high fat diet. As MSCs and MSC-EVs are known to have immunomodulatory capabilities we determined their effects on the expression of the inflammatory biomarkers in endpoint serum. Heatmap visualization indicates that MSC-EVs treated animals have the lowest normalized biomarker levels (Fig. 4A), suggesting generally lower inflammation levels in this group and thus a favourable immunomodulatory effect on a systemic level. However, there was no significant differences between the treatment groups for individual analytes (Additional file 1: Fig. S2). As synovial inflammation is one of the key components in OA development, we also measured the joint levels of iNOS and MMP-13, as indicators of inflammation and degeneration. MSC treatment induced the highest, and MSC-EV treatment stimulated the lowest MMP-13 expression in cartilage, as shown by MMP-13 histology staining. Although, compared to the vehicle group these differences were not statistically significant (Fig. 4B, D). Likewise, MSCtreatment increased synovium iNOS levels (Fig. 4C, E, *p* = 0.03), whilst MSC-EVs did not, indicating that MSCs induced the opposite effect to the immunomodulation favouring anti-inflammatory response reported in the literature so far [5, 6]. Together, these data indicate that MSC treatment increased synovial inflammation and cartilage degeneration markers, while MSC-EVs did not.

Body mass and metabolic disturbance can significantly affect the outcome of the OA, through promoting inflammation via metabolic modulators, such as the adipokine leptin [36]. To test if MSC and MSC-EV treatment affected the metabolism in our mild OA model, we assessed changes in body mass and metabolic disturbance. Body mass increased in all groups (Additional file 1: Fig. S3A). At week 11, 1 week before surgery, body mass and the variance of body mass was similar between groups (Additional file 1: Fig. S3B, *p* = 0.94 and Bartlett’s *p* = 0.73). At week 24, body mass in the MSC and MSC-EV treated groups showed greater variance then in the vehicle group (Additional file 1: Fig. S3C, *p* = 0.31 and Bartlett’s *p* = 0.036), indicating the variation between animals increased in the MSC and MSC-EV treated groups during and after the treatment period. Leptin concentration increased with increasing body mass in all treatment groups (Additional file 1: Fig. S3J). Importantly, leptin concentration was highest in MSCtreated rats, although not statistically different from MSC-EVs and vehicle groups. This might serve as an additional indication of the pro-inflammatory action of injected MSCs. Metabolic parameters such as HOMA-IR and triglyceride were the same in all treatment groups (Additional file 1: Fig. S3G–I).

In summary, these data suggest that in metabolic OA model, treatment with MSCs promotes the pro-inflammatory systemic effects, while MSC-EVs do not.

## Discussion

MSCs and MSC-EVs are promising disease modifying treatments in OA. They have immunomodulatory and regenerative properties, which could enable them to restore the imbalance between anabolic and catabolic processes in an OA joint [4–6, 9, 13, 14, 37]. Here, for the first time, we compared MSC and MSC-EV treatment in a mild OA model with disturbed metabolic context. We show that administration of MSCs has negative effects on the joint in a metabolic mild OA, which is an essential finding for metabolic OA phenotype patients represented by this model. In addition, our data suggests that the use of EVs as an injection therapy for OA might be more beneficial than the use of their parental MSCs. However, MSC-EVs had no significant therapeutic effect in their native, unmodified state.

The negative effect of a single injection of 2 million MSCs on OA progression reported in this study has not been picked up in any other OA model. In contrast, in majority, if not in all those studies, MSCs had a beneficial effect on cartilage regeneration [38–43]. The negative response to MSCs we observed, namely an inflammatory and painful OA phenotype with increased osteophytosis, might lie in the nature of the OA model used here. The high fat diet groove model is a mild and relatively slow (24 weeks) OA model, where the diet program clearly stimulates inflammation [24]. In other models, where often fast and excessive cartilage damage quickly leads to more severe forms of OA [44, 45], such a negative effect of MSCs could have been missed. On the other hand, it is also possible that the detrimental effect of MSCs is a consequence of disturbed metabolic environment in our model, under which normally beneficial MSCs can change their behaviour [46, 47]. In this light, our results might help to understand why translation of MSC treatment to the clinic not always resulted in good therapeutic efficacy, in contrast to the promising results found in animal studies [48]. Some randomised clinical trials report beneficial effects of MSC-based therapy for OA [49–51], while others find no effect [52, 53]. These clinical results combined with the findings in this study indicate that MSC treatment of OA might have more limitations than originally thought, and may even induce negative effects in certain situations. These are important drawbacks to consider when translating MSC treatment to the clinical practice, and possibly this is especially relevant when treating the metabolic OA phenotype. The high fat diet groove model might be more representative of the metabolic OA patients, who have a slow but progressive disease phenotype with a systemic inflammatory component. This group of patients might therefore need a different treatment approach.

Despite the high sensitivity for inflammation of the high fat diet groove OA model, MSC-EV treatment did not induce inflammation or joint degeneration. MSC-EVs are known to be less immunogenic than their parental cells, which is due to lower expression of surface proteins such as major histocompatibility complexes [54]. This can explain the lower synovial inflammation in response to repeated injections of MSC-EVs compared to a single injection of the parental cells, and as consequence lower osteophyte formation and pain behaviour. Synovial inflammation is known to aggravate osteophyte formation, via TGF-β and BMP production, by pro-inflammatory macrophages in the synovial lining [55, 56]. Likewise, inflammatory mediators can act on the peripheral nerves and induce pain [57]. MSC-EV injections did not induce pain-associated behaviours, even though the EVs were injected five times, to compensate for their fast degradation in the joint. The MSCs were injected only once and induced pain-associated response and inflammation. However, some limitations have to be taken into account here, as we have no quntitative data on the amount of EVs secreted in vivo by the MSCs, we cannot assume that the number of injected EVs was equal to amount of EVs released by the cells. Furthermore, we did not trace MSCs and MSC-EVs after injection in the joints, thus we do not know how similar their location and retention time was. Likewise, we were not able to establish whether the MSC-EVs and EVs secreted in vivo by the MSCs were endocytosed by other cells, such as fibroblasts, macrophages or chondrocytes in our OA model, which could have helped to elucidate a potential mechanism of action in the joint.

Little is known on the in-depth molecular mechanisms driving the effects of MSC and MSC-EV treatment in a metabolic OA setting. We made an attempt to unravel this and analysed a large panel of inflammatory and metabolic factors, which could possibly be regulated by MSCs or MSC-EVs. Nevertheless, this approach did not uncover any obvious candidates, that would explain the different effects following MSC and MSC-EV treatment in this study. Further and more detailed investigation of molecular signatures of MSCs and MSC-EVs in our mild metabolic OA model is necessary to better understand the underlying mechanism of both treatments.

Although MSC-EVs did not show clear therapeutic effect, our data suggests that the use of EVs as treatment for OA may be more beneficial than the use of their parental MSCs. MSC treatment increased synovial inflammation, measured by OARSI synovial inflammation score and iNOS positive staining as a marker for pro-inflammatory macrophages. Although MSC administration did not increase systemic inflammation markers in the serum as measured by multiplex assay at the end of the study (Fig. 4A and Additional file 1: Fig. S2), the activation of the immune cells resulting from the interaction with MSCs cannot be completely excluded. It is possible that the activation of immune cells and consequently changes in the levels of inflammatory markers have occurred at earlier time point and therefore we have missed it in our analysis. In contrast to single administration of MSCs, repeated injections of MSC-EVs induced overall lower synovial inflammation, osteophyte and pain response compared to MSCs. Similarly, some outcomes such as levels of systemic inflammation markers, levels of MMP-13 in the cartilage and cartilage mineralization show a trend of improvement following the MSC-EV treatment.

Local injection of human or other xenogeneic MSCs in OA models is often used and safe in immunocompetent rats and mice [38–42, 58]. However, the human source of MSCs used in this study might still be considered a possible explanation for the inflammatory response observed. Nevertheless, others have shown in a more severe rat OA model that cartilage regeneration was similar after treating the animals with human and rat MSCs [41]. Human clinical studies using higher dosages of 75–150 million allogenic MSCs reported more injection site pain and swelling [52], so dosage might also contribute to inflammatory responses. However, our study used 2 million MSCs, which is comparable to dosages of 1–5 million MSCs used in other rat OA studies where no inflammatory effects were reported [42, 59].

MSC-EVs are investigated as a promising treatment for OA in several preclinical models. They were found to inhibit inflammation, inhibit apoptosis of chondrocytes, promote regeneration and attenuate OA development [16–20]. In our model, however, we did not find a clear beneficial effect of MSC-EVs treatment. This might be a consequence of the relatively mild character of OA with metabolic component induced in our model. As previously shown, the high fat diet creates an ongoing inflammatory response when combined with cartilage damage [23, 24] and can reflect an important group of patients that have a chronic low-level obesity-induced systemic inflammation. In OA models without a dietary component, inflammatory responses might be more restricted to the locally induced damage [16–20], resulting in better EV therapeutic activity. However, MSC-EV-based therapy seems harder to translate to our high fat diet model where the systemic metabolic dysregulation and chronic inflammation will also influence OA outcome. Possibly, different strategies targeting specifically the low-level systemic inflammation may be needed here to treat the metabolic OA phenotype. As OA is a multifactorial disease and difficult to treat, combination therapy might be a solution here. MSC-EV-based therapy could be an addition to other promising pharmacologic approaches, such as sprifermin, tanezumab and IL-1β receptor antagonists, or other regenerative approaches, such as autologous chondrocyte implantation and use of scaffolds and gels for delivery [60].

The short half-life of EVs is a limitation of EV therapeutic effectivity. When EVs are administered systemically, the half-life is estimated at less than 6 h and they accumulate in the liver and spleen [61]. In this study, we injected both MSCs and MSC-EVs intra-articular, this is hypothesised to increase bioavailability while reducing systemic exposure, compared to systemic administration [62, 63], however very limited data is available on the differences in pharmacokinetics between MSCs and MSC-EVs when injected intra-articular. Systemically EVs are known to have a high degradation rate and clearance [61, 64, 65], to account for this we injected MSC-EVs five times, with 5 day intervals, however that did not result in significant therapeutic efficacy. To increase the therapeutic efficacy of MSC-EVs in OA, also in mild models such as ours, use of engineered EVs that accumulate longer in the joint or overexpress beneficial molecules, such as certain miRNAs, might be a future perspective. The miRNAs were demonstrated to play important role in EV-induced effects, which makes them an attractive target for EV engineering [66–70]. MSC-EVs carrying miRNAs such as miR-21a-5p, miR-146a, miR-199a, and miR-223 can control inflammatory genes and induce macrophage polarization towards an anti-inflammatory phenotype [67, 71]. Likewise, miR-214-3p is implicated in skeletal health and holds therapeutic potential to regulate bone formation [72]. By engineering EVs to carry specific miRNAs or other regulatory and pro-regenerative factors, EVs could be designed to inhibit pro-inflammatory effects of joint residual macrophages or to directly promote cartilage regeneration [66, 68–70, 73, 74]. However, to eventually bring EV-based treatment to the clinics, it is evident that better understanding of EV heterogeneity and working mechanisms are needed.

## Conclusions

In summary, our data demonstrate that MSC treatment has negative effects on the joint in metabolic mild OA, which is a crucial finding for design of future therapy targeted at the important group of patients with metabolic OA phenotype. Our results also suggest that MSC-EV-based treatment might be a more beneficial option for these patients, however it needs to be improved to increase the efficacy of the therapy, as in this study we demonstrated no evident beneficial effect of MSC-EVs on OA.

## Supplementary Information

**Additional file 1: Fig. S1.** Characterization of extracellular vesicles derived from human bone marrow MSCs. **A** MSC-EVs are positive for exosomal markers CD63 and CD9. EVs isolated from conditioned medium derived from primary bone marrow MSCs were subjected to sucrose density gradient followed by Western blot analysis for presence of CD63 and CD9. Representative Western-blots cropped at the molecular weight of CD63 and CD9 respectively are shown. **B** Nanosight Particle Tracking Analysis derived from 5 measurements showing that the nominal size of these vesicles is 125 nm. Red colour indicates error bar of the mean. **Fig. S2.** Inflammatory serum markers. Results of the 27 multiplex-assay showing all measured cytokines and chemokines from week 24 serum. One animal was excluded from multiplex analysis due to out of range values. Several EGF data points were excluded from analysis due to out of range values. Graphs represent mean ± 95% confidence interval, *p* values in graphs are reported with asterisks where *p* ≤ 0.05 is *, *p* ≤ 0.01 is **, *p* ≤ 0.001 is *** and *p* > 0.05 is not significant. **Fig. S3.** Body mass and metabolics. **A** Body mass between week 11 and 24 in the experimental protocol. **B** Body mass individual values at week 11 and **C** at week 24. Body mass increase as a percentage from week 11 in individual animals for **D** PBS, **E** MSCs and **F** MSC-EVs groups. **G** Homeostatic Model Assessment for Insulin Resistance, **H** triglyceride and **I** leptin concentrations. **J** Correlation between body mass and leptin concentration. Three animals were excluded from HOMA-IR analysis and one animal was excluded from leptin concentration analysis, due to out of range values. No animals or data points were excluded from all other analysis shown here, n = 12 per group. Graphs represent mean ± 95% confidence interval, *p* values in graphs are reported with asterisks where *p* ≤ 0.05 is *, *p* ≤ 0.01 is **, *p* ≤ 0.001 is *** and *p* > 0.05 is not significant. **Fig. S4.** Full length gels used in the Fig. S1A.

## Data Availability

The dataset supporting the conclusions of this article is available in the Open Science Framework repository, osf.io/mt2yq. Warmink, K. (2022, September 21). Mesenchymal Stem/Stromal Cells-derived extracellular vesicles as a potentially more beneficial therapeutic strategy than MSC-based treatment in a mild metabolic osteoarthritis model. Retrieved from https://osf.io/mt2yq

## Abbreviations

α-MEM: α-Minimal essential medium
µCT: Micro computed tomography
ATMP: Advanced therapy medicinal product
BV/TV: Bone volume/tissue volume
CCMO: Central Committee on Research Involving Human Subjects
DWB: Dynamic weight bearing
EV: Extracellular vesicle
HOMA-IR: Homeostatic model assessment for insulin resistance
iNOS: Inducible nitric oxide synthase
MMP-13: Matrix metalloproteinase 13
MSC: Mesenchymal stromal/stem cell
MSC-EV: MSC-derived extracellular vesicles
OA: Osteoarthritis
PBS: Phosphate-buffered saline
PL: Platelet lysate
RL/RR: Rear left/rear right
ROI: Region of interest
TNF: Tumor necrosis factor
