# Supplementary figures and images for: Mesenchymal stem/stromal cells-derived extracellular vesicles as a potentially more beneficial therapeutic strategy than MSC-based treatment in a mild metabolic osteoarthritis model
Source: Stem Cell Res Ther. 2023 May 24;14:137. doi: 10.1186/s13287-023-03368-7 (PMC10210425; doi:10.1186/s13287-023-03368-7)

**A**

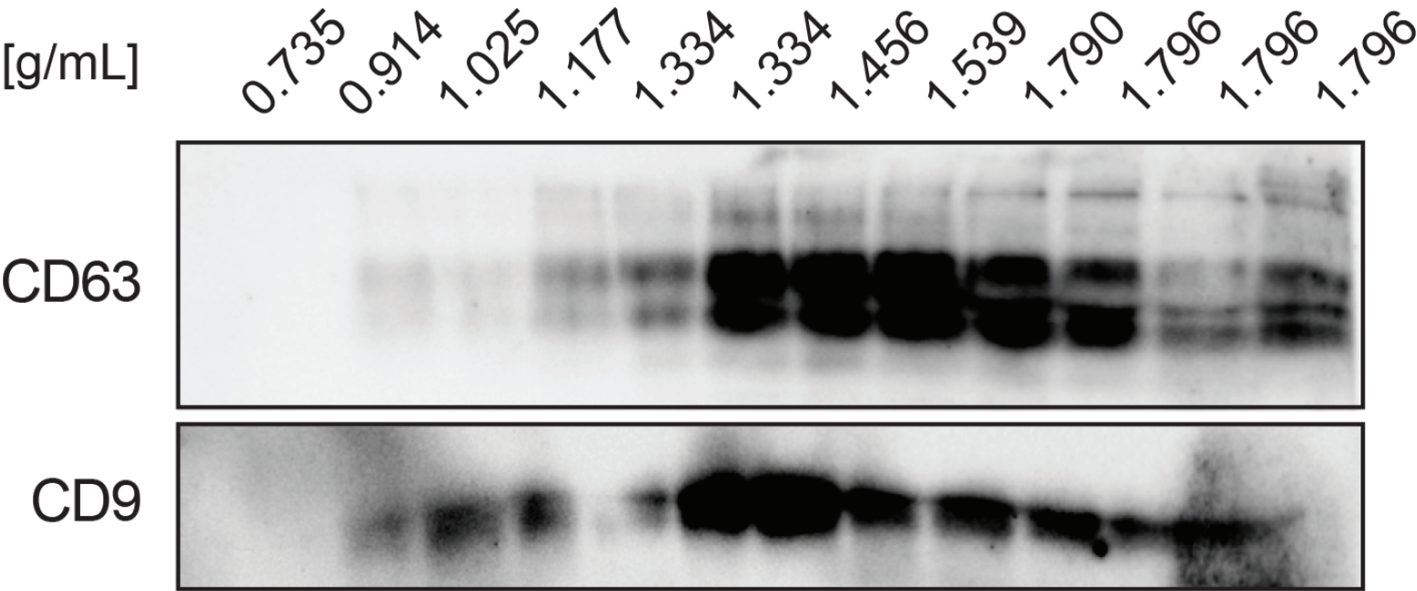

**B**

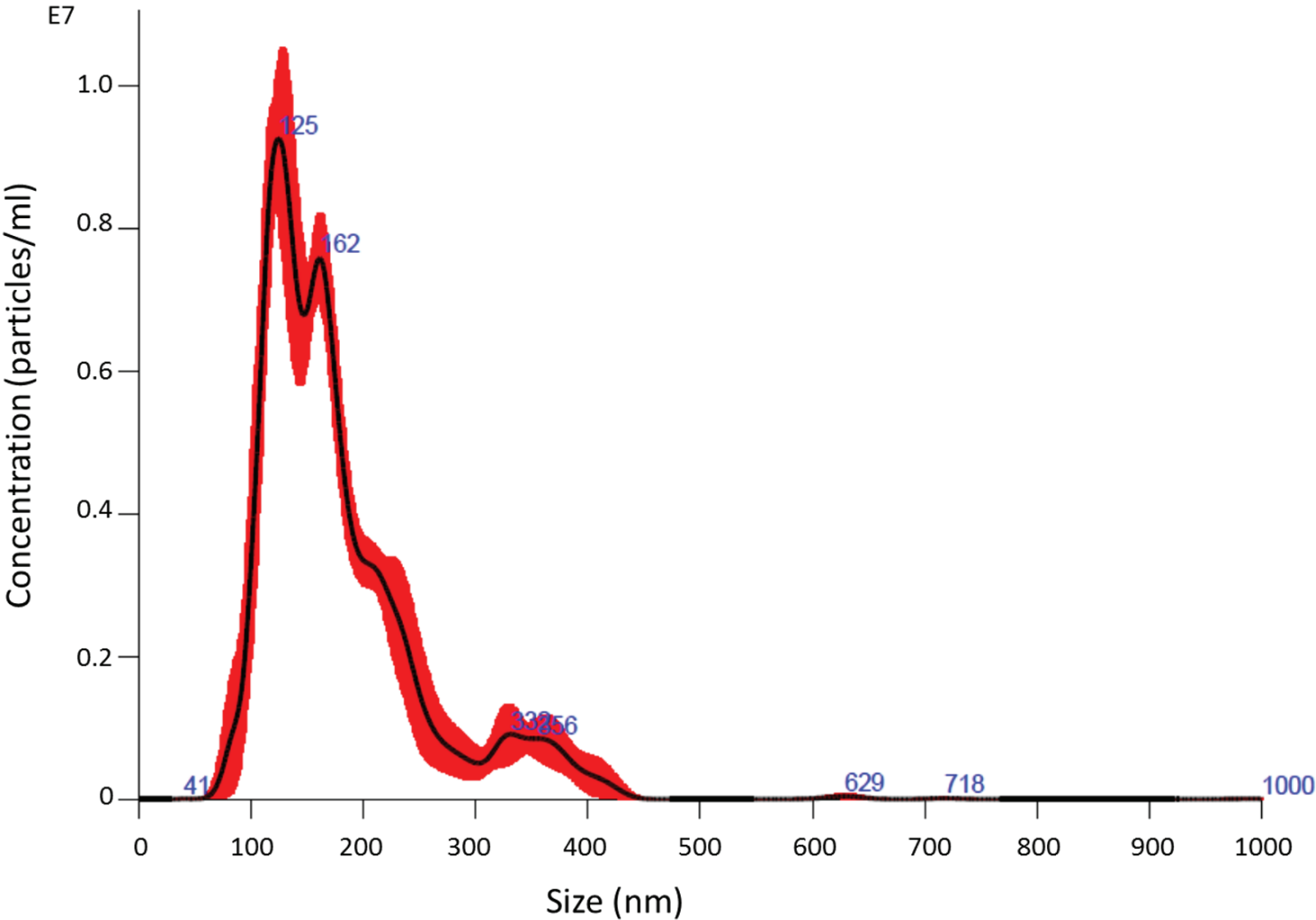

# Supplementary Figure 2

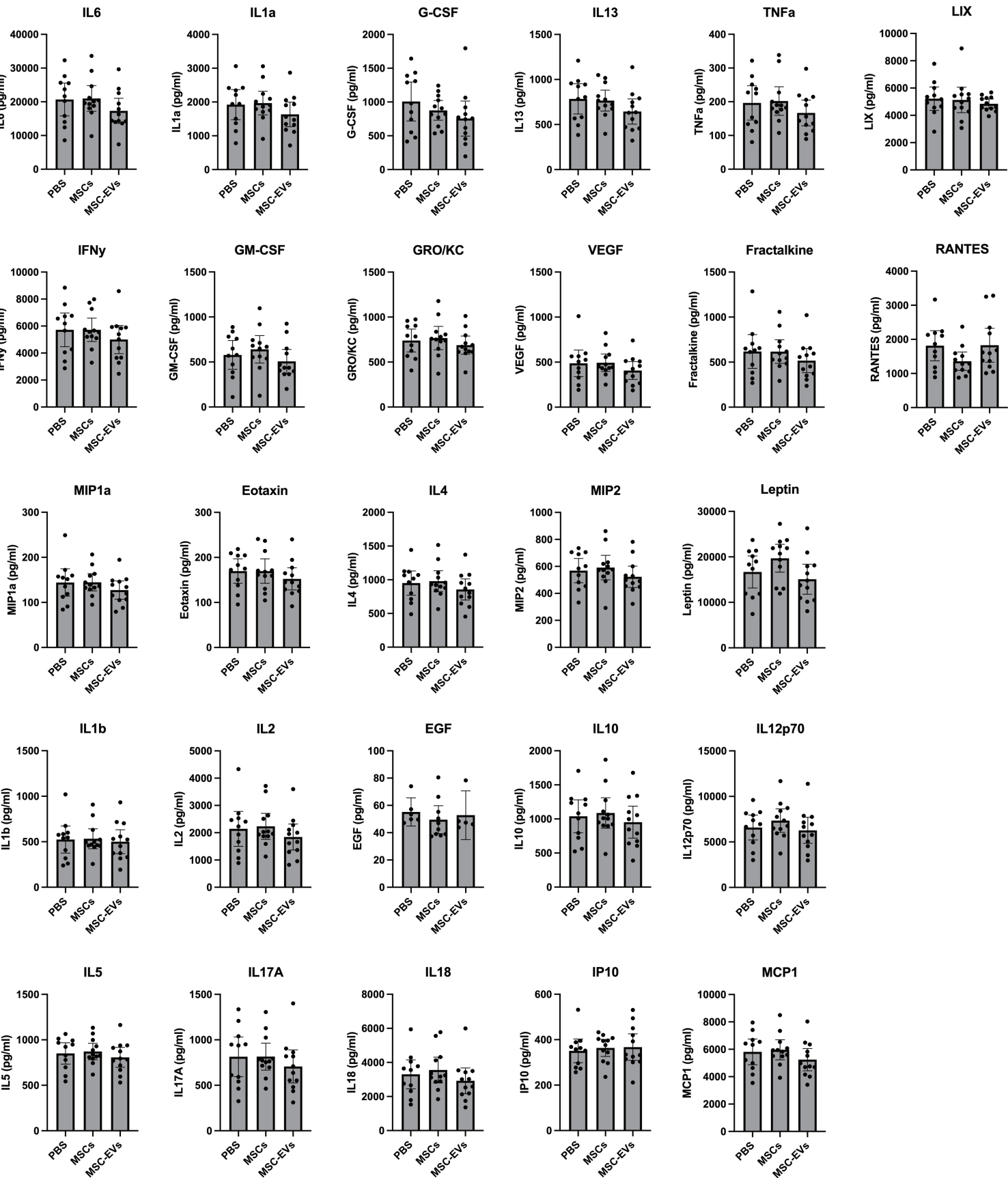

# Supplementary Figure 3

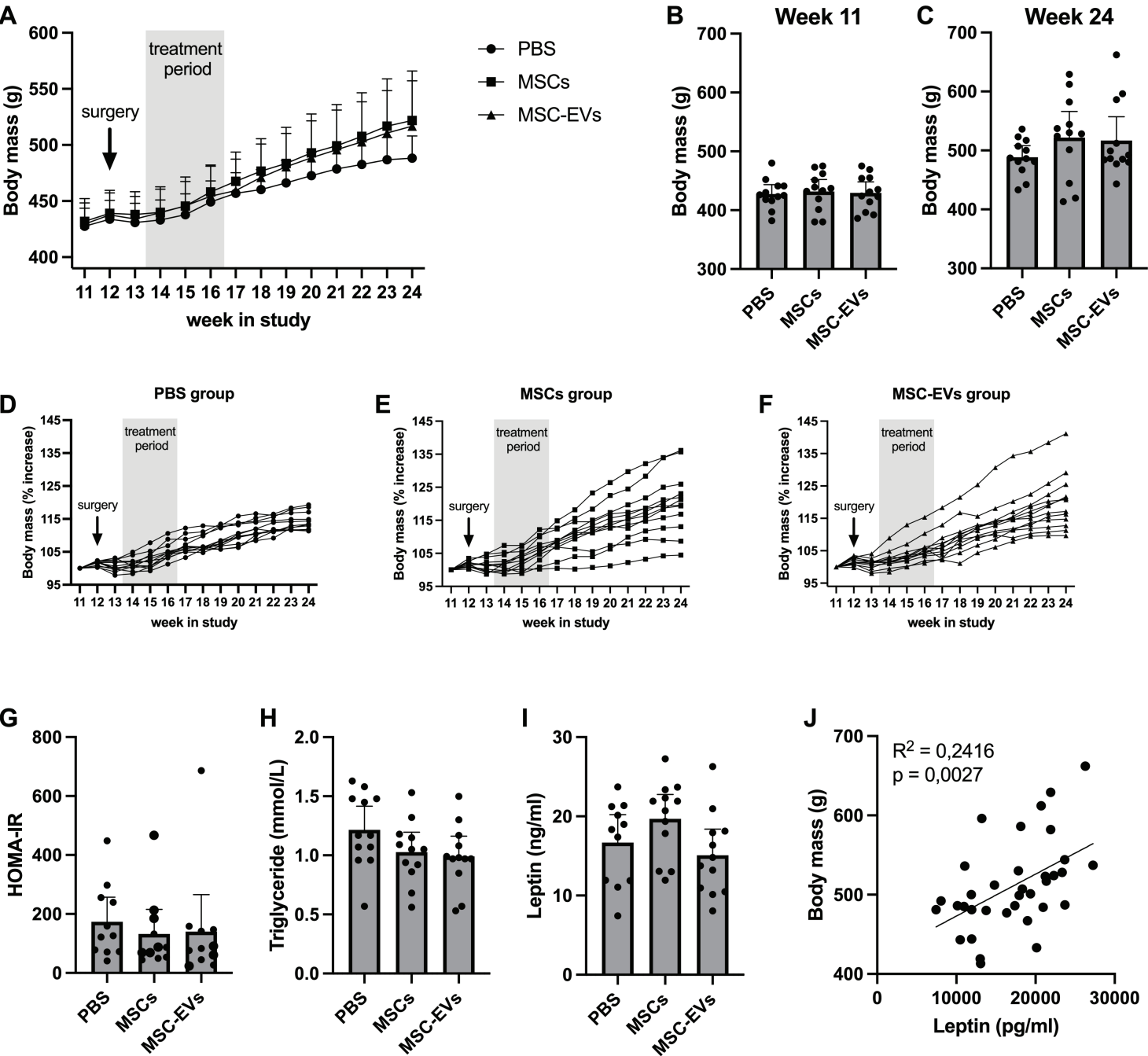

Supplementary Figure 4

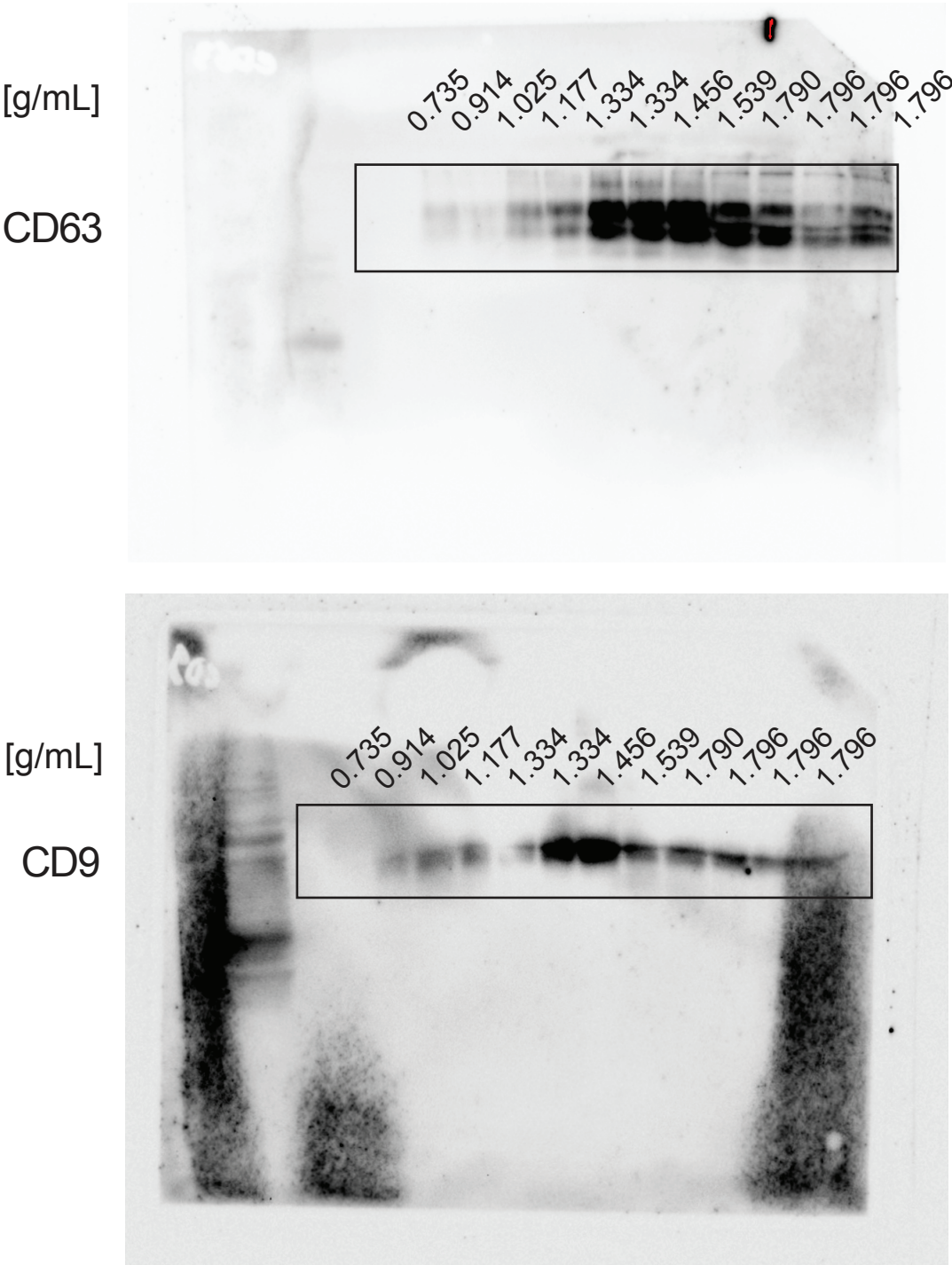

Supplement: Supplementary file 1 — Additional file 1: Fig. S1. Characterization of extracellular vesicles derived from human bone marrow MSCs. A MSC-EVs are positive for exosomal markers CD63 and CD9. EVs isolated from conditioned medium derived from primary bone marrow MSCs were subjected to sucrose density gradient followed by Western blot analysis for presence of CD63 and CD9. Representative Western-blots cropped at the molecular weight of CD63 and CD9 respectively are shown. B Nanosight Particle Tracking Analysis derived from 5 measurements showing that the nominal size of these vesicles is 125 nm. Red colour indicates error bar of the mean. Fig. S2. Inflammatory serum markers. Results of the 27 multiplex-assay showing all measured cytokines and chemokines from week 24 serum. One animal was excluded from multiplex analysis due to out of range values. Several EGF data points were excluded from analysis due to out of range values. Graphs represent mean ± 95% confidence interval, p values in graphs are reported with asterisks where p ≤ 0.05 is *, p ≤ 0.01 is **, p ≤ 0.001 is *** and p > 0.05 is not significant. Fig. S3. Body mass and metabolics. A Body mass between week 11 and 24 in the experimental protocol. B Body mass individual values at week 11 and C at week 24. Body mass increase as a percentage from week 11 in individual animals for D PBS, E MSCs and F MSC-EVs groups. G Homeostatic Model Assessment for Insulin Resistance, H triglyceride and I leptin concentrations. J Correlation between body mass and leptin concentration. Three animals were excluded from HOMA-IR analysis and one animal was excluded from leptin concentration analysis, due to out of range values. No animals or data points were excluded from all other analysis shown here, n = 12 per group. Graphs represent mean ± 95% confidence interval, p values in graphs are reported with asterisks where p ≤ 0.05 is *, p ≤ 0.01 is **, p ≤ 0.001 is *** and p > 0.05 is not significant. Fig. S4. Full length gels used in the Fig. S1A. [file 13287_2023_3368_MOESM1_ESM.pdf]
